# “As young men we have a role to play in preventing sexual violence”: Development and relevance of the men with conscience intervention to prevent sexual violence

**DOI:** 10.1371/journal.pone.0244550

**Published:** 2021-01-07

**Authors:** Tania de Villiers, Sinegugu Duma, Naeemah Abrahams

**Affiliations:** 1 Division of Nursing and Midwifery, Department of Health and Rehabilitation Sciences, Faculty of Health Sciences, University of Cape Town, Cape Town, South Africa; 2 College of Health Sciences, University of Kwazulu-Natal, Durban, South Africa; 3 Gender and Health Research Unit, SA Medical Research Council, Women’s Health Research Unit, Faculty of Health Sciences, University of Cape Town, Cape Town, South Africa; University of Sao Paulo Medical School, BRAZIL

## Abstract

Sexual violence against women and girls is a major public health problem globally and in South Africa. Although young men have been identified as an important risk group for prevention interventions, scant attention have been given to this age cohort in low and middle-income countries. There is strong evidence that perpetration starts early and increasing attention is being drawn to Higher Education Institutions (HEIs) as settings for prevention interventions. The main objective of this study was to adapt the One Man Can Intervention for use with male university students in residences and develop materials for implementation. This paper presents the qualitative findings of the adaptation process of the One Man Can Intervention with 15 young male student leaders at a HEI in South Africa. The same participants who started in the study, participated throughout. Only five of the 15 participants were located and participated in the interviews six months post intervention. The results show the emergence of a six-hour session adapted intervention that addresses key drivers of violence against women and girls (VAWG). Critical engagement and dialogue on sexual violence is shown to shift key norms on gender equality, on being a man and reflection on their role in preventing sexual violence. This paper contributes to the field where much learning, refining and improvement of prevention interventions for VAWG are ongoing.

## Introduction

Sexual violence reported to the South African police shows 52 420 sexual offences were reported in the 2018–2019 reporting year, which relates to 114 per day and a rate of 72/100 000 population [1]. This is very likely an under count as under-reporting of sexual violence to police is well-known globally [2, 3]. These high rates are confirmed in a population study in Gauteng province which showed only 1 in 23 women who reported the sexual violence in the study, also reported to police [4].

Perpetration risk factors studies have emerged in the last few years and this has fed into the development of theory-based prevention interventions for men [5]. The literature on risk factors has clearly identified gender inequality as a key structural factor which together with poverty and acceptance of violence contribute to the normalizing of social norms on masculinity and acceptability of using violence against women and children [6]. Other drivers include individual and relationship level factors such as childhood exposure to violence, alcohol misuse and communication within relationships [7–12]. Many of these drivers are commonly found in South African society where dominant masculinities are shaped by the colonial past, a legacy of apartheid linked to experiences of political violence, all coalescing into violent masculinities which is further embedded in racial inequalities [13, 14].

The Sonke Gender Justice’s One-Man Can intervention is a theory-based, community-based, gender-transformative intervention developed in South Africa with a focus on encouraging men to engage in gender equitable relationships with women [15–17]. The original One Man Can intervention consist of 29 interactive, action-orientated workshops and responds to both GBV and HIV risks. Evidence from qualitative research show promising outcomes and together with good practice from primary prevention interventions from other parts of the world i.e. the United Nations Populations Fund (UNFPA) and the Humanitarian Crisis Settings in Sub-Saharan Africa [15, 18] provides impetus for this intervention to be adapted for different settings.

Higher education settings have not received the needed attention for prevention interventions. A review of the literature including grey literature showed no sexual violence prevention interventions within the South African higher education settings by 2017, although numerous reports of sexual assaults involving students and staff as perpetrators have been made [10, 19]. In view of the latter, sexual violence in university settings in South Africa has become a key part of students’ responses and activism in the face of poor transformation. This study originated at a time when sexual violence perpetration and victimization in South Africa made media headlines almost on a daily basis, creating mass responses within university communities across the country. The fees must fall activism across South African universities also raised the issue of sexual violence within campus settings and this provided impetus to initiate prevention of sexual violence within Higher Education settings in South Africa. The urgency of addressing sexual violence prevention was recognized as an important directive by the South African Government. Although many feminist and community organizations have lobbied against sexual violence, and social welfare and judicial structures were in place to address this issue, there were no clear guidelines on how to address prevention of sexual violence within university settings and sexual violence in South African universities remains a problem.

Many robust studies have been conducted on prevention of GBV, which included intimate partner violence and sexual violence, and tested in randomized control trials in South Africa, but these were largely community based. For example, the Stepping Stones intervention with young men and women to prevent HIV infections [20] and Sonke’s One Man Can Intervention [16]. The urgency to implement prevention interventions with men at universities is now more relevant than ever before and the outcomes of this study aimed to address this gap. This paper presents the adaptation and implementation process of the OMC intervention for University settings. The iterative adaptation process resulted in the ‘Men with Conscience’ intervention which identified six core workshops and included assessing the feasibility of delivering the intervention to male students in HEI settings.

## Methods

### Study design

A case study design was used to conduct this study, because it enabled the study of a real-life phenomenon, within a specific time frame, in a specific location and with a specific group of people [21]. The study used a number of qualitative data collection methods which included two focus group discussions (pre and post intervention), five semi-structured interviews with participants six months after intervention delivery, discussions from the five intervention workshops, open-ended questions completed by the young men at the end of each workshop (participants’ reflections) and researchers’ field notes from observation during the intervention workshops. The main adaptation was to shorten the number of workshops as students would not attend if it happened over a long period while still focusing on key social norm change. The data from the first FGD were used as a guide, which was compared to the workshops used in the original One Man Can Intervention. The comparison was done to see if adaptation was necessary and if so, which aspects of the original One Man Can Intervention workshops needed to be adapted. Data from the first FGD thus informed implementation of workshop one.

The adaptation followed an iterative process of choosing the follow-up workshop on what emerged in the previous one (see S1 Appendix).

The format of the five intervention workshops was similar to focus group discussions, but included participatory methodology commonly used and found to be effective in prevention interventions across disciplines [22]. These workshop discussions were therefore considered as data, because it showed how the young men engaged with the intervention topics (see Table 1).

**Table 1:** Description of the workshops conducted.

Objective	Details on adaptation
Pre-workshop Focus Group Discussion	To assess participants’ knowledge and understanding of sexual violence within a broader society and the university context.
Workshop 1: Addressing personal values and belief systems	To explore values and attitudes around sexual violence and men.
Workshop 2: Societal pressures for men’s behavior	To recognize that it could be difficult for men to fulfil their gender roles that are present in society’
Workshop 3: Defining Rape	To discuss consensual sex.
Workshop 4: Courage to act	T*o* explore men’s perceptions of their degree of courage in bystander intervention.
Workshop 5: Maintaining healthy relationships	To be able to identify healthy vs and unhealthy relationships.

The research setting was undergraduate male residences at one university in South Africa.

### Sampling and participants

*The sample consisted of 15 male student leaders*, 11 of whom were Black & 4 White. All names of participants stated in this paper are psuedonyms, which were randomly allocated according to the racial classification. Reference to race is made in the context of the legacy of apartheid and the racial classification system used in South Africa. The racial profile of the sample is reflective of the students in the residences from second year onwards in South Africa, where most students who can afford to, move out to alternative accommodation, such as their own apartments or sharing private accommodation near campus. The economic profile of participants ranged from low to high income students. For example, students who come from affluent backgrounds, would move into their privately-owned accommodation, which is more expensive than the university residences, while some students who are from poor socio-economic backgrounds, and dependent on the National Student Financial Aid Scheme (NSFAS) bursary, often remain in student residences.

Purposive sampling was used to select participants. The study participants were male university student leaders in residences. This was the first study of its kind in university settings with a specific focus on university residences, where sexual violence was mostly a problem. Male student leaders in residences were purposefully selected and used as key informants, because of the position they held in the residences and the influence they could have in the prevention of sexual violence in the residences if the adapted OMCI was successfully implemented. We purposefully did not include first year students since we wanted discussions on experiences within the student residence systems and post graduate students are often not in residences anymore.

### Enrolment and ethics

Ethical clearance to conduct this study was granted by the Health Sciences Ethics Committee in the Faculty of Health Sciences at the university where the study was conducted in South Africa. Permission to access student residences was granted by the university’s Director of student housing. All participants were assured of anonymity in reporting the research findings.

Research assistants visited the identified male student residences to inform and invite student leaders in these residences to participate in the study. The latter was followed up and confirmed by phone calls, emails and text messaging. Participants who agreed to enroll in the study, were asked to complete the consent form, after which they were invited to the first FGD which focused on sexual violence in university residences. The recruitment phase was critical to the success of the study, which required commitment, support and ongoing communication from the various residence structures. The sample size was 15 male student leaders with ages ranging from 20 to 25 years. Participants were second to fourth year students studying across different faculties i.e. humanities, commerce, law, engineering and health sciences. The same participants who started in the study, participated throughout. Only five of the 15 participants were located and participated in the interviews six months post intervention.

### Data collection

Data was collected from mid-August to end October 2014.

**The pre-intervention focus group discussion** with 15 participants was used to assess participants knowledge and understanding of sexual violence within a broader society and within the broader university context. This pre-intervention FGD became the first workshop of the adapted intervention. The topic of the next intervention workshop was decided on by the team after examining what emerged in the FGD (see Table 1 for the list of workshops and topics). The research team met after each workshop and together decided which of the OMC workshops to use or to adapt for the needs of the group and also which participatory methods to use at the next workshop. Developing themes from the pre-intervention FGD was critical as it informed the structure of workshop one. It also determined the kind of adaptation of the original One Man Can Intervention for workshop one that was needed. Development of each workshop was dependent on what emerged in the previous workshop. The post intervention focus group discussion after the six workshops with 15 participants was to determine if there was any change in perceptions, if learning happened, or if participants found the workshops’ content useful (see S2 Appendix for the questions that were asked in the post-intervention focus group discussion).

**Research field notes** were collected during the FGD and intervention workshops by the research assistants. These included the delivery process and implementation of the five workshops, observations included what worked well, what did not work, attendance, achievement of objectives and possible improvements. Fieldnotes also captured non-verbal or unspoken elements in the research process such as reactions to statements as well as obvious emotions such as surprise, contempt and sometimes disgust. The research notes were combined with the transcribed workshop discussions data. The open-ended questionnaire completed by participants at the end of each intervention workshop which included their reflections of the workshop was also included as data. Examples of the open-ended questions were “*What did the session mean to you today*?*”; “Were there any aspects of today’s session that resonated with you*? *If so*, *explain why”; “If the session did not resonate with you*, *explain why”*.

#### Semi-structured interviews

Semi-structured interviews were conducted six months after delivery of the adapted intervention with five students who were contactable and available. Most of the participants had either graduated and were no longer students at the university, moved to different accommodation facilities or were not available during the time period when the interviews were conducted. The objective for conducting the semi-structured interviews was to qualitatively assess how participants responded to the intervention after a six-month period, i.e. how much of the learning they retained and applied in their daily lives.

Members of the research team included the lead interventionist who had extensive experience on working with men on the original One Man Can intervention at the Sonke Gender Justice Program as well as the study PI (1^st^ author) and research assistants. The lead Interventionist chose co-facilitators, as this is the format of the One man Can intervention [16]. A central and conveniently located venue was used for the duration of the study. Workshops were conducted in English and each FGD lasted approximately 60 minutes. Retention in the workshops was influenced by the mid-term university vacation, where some students visited their families who lived far away from the university location, hence not everyone attending all six workshops.

### Data management

All data obtained from the different data collection processes were coded and labelled, i.e. no names of any of the participants were revealed. The authors were the only ones with access to the codes, thus protecting the identity of each participant. Data were stored safely and securely. After transcription, data was entered using the NVivo qualitative data analysis software program, which assisted with data management.

### Data analysis

Data analysis sought to piece together the strands from the different data sources into themes, and by so doing present a detailed interpretation. The themes that emerged from the pre-intervention FGD were used as a guide, which was compared to the workshops used in the original One Man Can Intervention. Observations, students’ weekly reflections, the post-intervention FGD and the semi-structured interviews were transcribed verbatim and analysed using the thematic data analysis technique. The final outcome of the data analysis, which is presented in this paper, w*as* an intervention for use in university settings named the Men with Conscience (MWC) intervention (see Table 2).

**Table 2:** Summary of themes adapted from the OMC to the adapted men with conscious intervention for the university setting.

Number of workshops	OMCI	Adapted OMCI (Men With Conscience)
Workshop 1		Locate sexual violence in context
Workshop 2	Gender Values clarification	Personal values and belief systems
Workshop 3	Act like a man, act like a woman	Societal prescriptions for men’s behaviour
Workshop 4	Consent vs Coercion	Defining Rape
Workshop 5	New Kinds of Courage	Courage to act
Workshop 6	Defining the ideal partner	Maintaining your own identity in intimate relationships

## Findings

### Discussing sexual violence

The study found sexual violence was not discussed within the University setting and the pre-intervention focus group discussion provided them with their first frank discussion of sexual violence with their peers. Many agreed “**… *by talking in a comfortable way is where we will find solutions”* [Khagiso, third-year Commerce student].** However, participants recognized the discomfort in discussing sexual violence and the role of prevailing social norms that prevented discussions of private and sensitive issues. The young men recognized early in discussion that the absence of information on all aspects of sexual violence was needed to find solutions as one said this is where **‘issues around how we can prevent sexual violence”** will emerge. They also identified missed opportunities for sexual violence discussions and said: *‘****People don’t know where to go for information on sexual violence*, *because there’s no real emphasis on sexual violence during O’ week****”* **[Dzimba, a second-year Law student].** The reference to O’ week which refers to the orientation week for first year students emerged often across the discussions as both an opportunity to share information but also a time of heightened perpetration of sexual violence by male students on vulnerable female students.

### Conquests (sexual) as part of being a man

Their recognition of how social norms impact on male behaviours, particularly male sexual behaviour and being a successful man, was a recurring theme. A participant explained *“****We all have sex (men and women)*, *but there’s more expectations on men to have sex than on women*, *like in O’week guys in res count how many girls you had*. *The more girls you have to your count the bigger the man you are”*. [Morgan, third-year Commerce student].** These young men clearly affirm how gaining status from peers through demonstration of manhood through sexual conquests occurred, which has been reported in numerous studies on masculinities.

[23–29] occurred in the university setting. The resultant pressure to meet expectations created struggles within the young men and they revealed how they would rather lie about having sex with many girls than admitting not having any sexual encounters with girls. Many agreed with giggling affirmations to “***Yeah … it’s crazy but it’s true*. *I’d rather not say anything about not scoring* (**not having sexual conquests**), *r*ather *than to be ‘mocked’ by guys or be seen as less than a man*. *It’s like there’s something wrong with you”* [Khagiso, a third-year Commerce student].**

The young men were telling each other what the norms were that they should subscribe to, the associated pressure to conform and the demands on them to demonstrate success as a man through sexual prowess. Referring to themselves as being part of the male group they gave examples of masculine norms and said: “***For us as young men*, *we feel strongly that men should be heterosexual … meaning he must have a woman and have children … have a family that he can provide for*, *that’s important****”* ***[Jabu]*.** *Another said “****As a group*, *we felt that a man must be the provider of his family … he must protect his wife and children”* [Sitole].**

The young men questioned and criticized the role of religion in promoting sexual violence. One student said *“****Religion and religious values also play an important part in sexual violence*, *especially if it’s between married people*, *e*.*g*. *religion says married couples should stay married*, *irrespective and just pray the problem away*. *To me it doesn’t work like that*! *I don’t believe God is so narrow-minded*, *because wrong is wrong anyway*!*”* [Jaye, second-year Social Science student**]. Many participants agreed with Jaye’s thinking that religion “**failed women**” through the reinforcement of sexual violence, by perceiving sex in marriage as the man’s “marital right”.

Soon after the pre-intervention FGD the team recognized and agreed that the topic of the pre-intervention FGD “locating sexual violence in context” should be incorporated as the first intervention workshop. The team further decided, given the discussion in the first FGD, to use the OMC workshop on Gender Values clarification for the second intervention workshop as this would allow the young men to explore individual and common values and attitudes around sexual violence and men.

### The personal

In the second intervention workshop known as ‘Personal values and belief system’, provocative statements were presented to participants to elicit discussions and debate, e.g. 1) “it is easier to be a man than it is to be a woman”; 2) “Sex is more important to men than to women”; 3) “a man is sexually aroused it is very difficult for him not to have sex” and “Women who wear short skirts and revealing clothes are partly to blame when they are raped”; to engage men on their understanding of their own value systems in relation to sexual violence and gender. The frank and open discussion of their perceptions, opinions and experiences related to gender-based violence, showed their commitment to have honest discussions and to seek to understand how male behaviour is rooted in societal norms. A student shared his childhood experiences of witnessing his father’s abusive behaviour towards his mother and how this behaviour became “**normal**” in their household. He said: *“****I remember growing up*, *how my dad used to beat up my mom if she didn’t do as he wanted*. *I grew up believing that’s how you treat a woman… seriously bro*! *Now I can think that maybe he forced my mom to have sex with him*, *because what if he wanted sex and she refused*. *He gives her a slap*. *I know this is personal*, *but let’s call a spade a spade dudes*? *As a boy you grow up seeing these things as normal*, *when in fact it’s not okay to beat on a woman or force sex****”*. **[Sitole, second-year Law student].** This recognition is evidence of their own understanding of violence against women overall and sexual violence in particular. The sharing of personal stories also indicated trust and comfort during the workshops. They also demonstrated their understanding of sexual violence as a serious problem in their families, intimate relationships and their student community.

### “Pressure on me”

Given their discussions on the personal struggles, the topic of the third intervention workshop was chosen to assist men to recognise that it could be difficult for them to fulfil their expected gender roles. They acknowledged how “**difficult**” it was to be a man and were critical of society and said: **“Expectations society has on me as man put pressure on me**…”. The same participant interrogated societal prescriptions of male behaviour and perceived society holding “**double standards**” by not teaching men how to take responsibility for their actions. They demonstrated their understanding of how rape myths are embedded in gender norms of sexual behaviours and used the blame afforded to women for being raped as an example of social expectations and double standards applied to men and women ‘…***Let’s say a girl walks past you dressed in skimpy clothing*, *society and men think that women are cheap right*? *… but if a guy walks without his shirt on he’s seen as macho and cool with his six pack*. *If a woman is raped*, *the first thing people ask is ‘What was she wearing’*? *I think that is wrong and that is where society and men are messed up*, *coz no matter what a person wears*, *there is no justification for raping a girl*!*”* [Sifiso, third-year Business Management student].** Furthermore, these discussions showed this workshop allowed the young men to use critical thinking to unpack their value systems related to sexual violence and provided evidence of the workshop achieving its goal.

The discussion on different moral standards for men and women merged into discussion of power and they gave their perceptions of how men, despite their abusive behaviour towards women, still gain respect because they are considered powerful. The local politicians were used as examples with a young law student *saying “****Many great men*, *like politicians were womanisers*, *but they were seen as heroes to society*. *Just look at our own president”* [Sitole, second-year Law student].** This was reference to the rape trial of former president Jacob Zuma who was acquitted while the victim was severely judged by most South Africans [30, 31].

Participants further discussed how “**bad role modelling**” fuels the problem of sexual violence and identified the absence of “**good**” male role models to teach them how to be “**real men**” and said this was a possible reason why “**men screw up**”, meaning men can mess up or make mistakes and still be perceived as hero’s, but the same does not apply to women who make mistakes. This discussion and critical reflection during this session, signified a shift in their personal views and evidence of change in attitude.

### “Wake-up call”

Participants’ realisation of the interconnectedness of sexual violence and interpersonal relationships became evident during discussions on consent in the fourth workshop. The discussion evolved into advisory type conversations among the men such as **“*Rape is real*, *and it happens in contexts that you never expect*. *Golden rule*, *never assume that sex is consensual and always wait for the ‘yes’ then you will be safe*.*”* [Alvin, third-year Engineering student].** Another senior student in this session said “***This session was definitely a wake-up call for me*! *I’ve realised that there is more to rape than just sex without consent*. *You actually really need to ask the person is she okay with having sex and she has to say ‘yes’ coz if she says anything else … dude … you’re a rapist”* [Mandla, third-year Engineering student].** During this session all participants verbalized and agreed on what consensual sex meant, and this evolved into their willingness to take responsibility for their own sexual behaviour.

This session on defining rape presented the young men with the reality of what encompasses rape. It was observed during this session that a critical turning point was reached when participants reflected on their previous behaviour and realised how their sexual behaviour with partners was wrong and more complex than they had thought initially. A young man said “***I carried on until she stopped saying ‘NO’ … I realise NOW it was wrong*, *because I didn’t hear the ‘YES’*. [Khagiso, third-year Commerce student].** In this workshop men started to realise they could have been guilty of rape at some point in their lives. Participants reflected on their own sexual behaviour to identify unacceptable sexual conduct towards women in the past. They valued the full understanding of sexual violence and rape and its legal frameworks. However, the participants raised concern that the poor understanding of sexual violence and rape was widespread within the University and said *“****Students … are not necessarily knowledgeable according to the law*.*”* [Dzimba, second-year Law student].**

After the completion of the project it came to the researcher’s attention that the participants extended the discussion beyond the workshops into the residence dining halls and social media such as Facebook, Twitter and WhatsApp. They shared the information they had learned in this session with others. The disconnect between the legal definition of sexual violence and the commonly understood meaning among students were one of the messages the young men shared, and this demonstrated evidence of change, taking responsibility and their commitment to help prevent sexual violence within the University residence and beyond. The impact of the intervention was most evident when the young men accepted accountability by publicly labeling themselves as “**rapists**” on social media. This public demonstration of their stand against sexual violence is another indication how participants transitioned towards change.

### Intervening

The last two workshops dealt with their role to prevent violence against women and how to maintain healthy, respectful relationships with women. The OMC workshop on “New kinds of courage” was adapted and named “Courage to act”. The men identified intervening in abusive relationships as a challenge and felt among others their courage to intervene was dependent upon the nature of the friendship, their own marital status, sexual orientation of the couple (i.e. homosexual or heterosexual), and religious values with the latter considered as most disempowering. Many agreed that ***“Religion says married couples should stay married”*.** *Another said “****The challenge that I have is that when married people have problems or you can see there is abuse going on right … if you intervene … let’s say I am not married but I intervene …they tend to say to the unmarried person like what do you know*, *you are not married*? *So that makes intervening in that case difficult”* [Morgan, third-year Commerce student].**

The consideration of courage in relation to homosexual men was linked to the participants’ poor knowledge of homosexual men and sexual violence. During the fifth intervention workshop it became clear that misconception of sexual violence among students of different sexual identities was important to be included as it is often not considered in the development of sexual violence prevention strategies in university residence settings.

Participants not only learned how to identify unacceptable behaviour by others, but that they were able to reflect on their own behaviour and know when to intervene. Based on the knowledge they gained on sexual violence throughout this series of workshops, they recognized the change in themselves, which enabled them to engage with the idea of intervening as bystanders. They felt that although bystander intervention may be difficult at times, it was very important to intervene when abuse was observed. They also felt that being knowledgeable about sexual violence, knowing when to intervene and understanding the nature and context of a relationship were important factors associated with bystander intervention. In identifying themselves as bystanders, participants reflected how they have taken ownership of the problem of sexual violence in university.

### Healthy relationships

The team decided that the intervention must deal with relationships and the OMC “Defining the ideal partner “was adapted to “Maintaining your own identity in intimate relationships”. The men recognized that respecting women is an important element in a healthy relationship and the theme of respect was a recurring theme in many of the workshops. Participants identified trust, respect and honesty as important ingredients to maintaining a healthy relationship, which could also assist in preventing sexual violence from happening. A young man said: ***“Respect is a big thing*, *in any relationship and should be a mutual experience*, *especially when it comes to sex”*.** Another student also linked respect in a relationship to sexual behaviour and again warned others of being a rapist if not respecting the wishes of a partner. ***“Respect is a big thing*, *in any relationship and should be a mutual experience*, *especially when it comes to sex*. *So*, *if your woman says she’s not in the mood (she does not want to have sex) then do not force her*, *but respect her wishes*, *otherwise you’re a rapist dude”* [Tselo, third-year Social Science student].**

Communication within relationships was therefore part of this last workshop and recognition of open communication about sex in a relationship was recognized. They further recognized that men and women could communicate their needs differently. A student advised, *“****There is a difference in the way men and in the way women communicate*. *Either way*, *both parties should be clear and honest enough with each other how they feel about things*, *like for example sex*. *Like Tselo said*, *if the woman is not in the mood*, *then tell her how you feel about her always not being in the mood makes you feel*. *In that way you don’t have to force things and then you are not committing sexual violence”* [Sitole, second-year Law student].**

Religion and culture were recurring factors identified as “**failing women**”. This was evidence that participants were piecing together the strands of the sexual violence tapestry, i.e. making connections to the interrelatedness and complexity of sexual violence perpetration, for example male dominance, men “failing women” and the impact of religious values. *“****I honestly think we give culture too much scope in our relationships and that can be a problem*. *I think that is why we have so many problems in our society*, *because culture and beliefs are often just black and white … there’s no room for taking each situation as it is*, *because what if I love a Xhosa girl but my culture says I can only get involved with a white girl*? *That’s just reality”* [Patrick, second-year Engineering student].**

There was often disagreement amongst participants throughout the workshops when they challenged each other’s views and perceptions. The team allowed this to happen in healthy ways and this experience reinforced the learnings of respect. It was clear from the observations of all the workshops that participants drew on previous workshops. This demonstrates some understanding they gained on the complexity of sexual violence and the interplay between the different contributing factors, such as joint decision making and clearly defined roles in a relationship.

Some participants felt that there was a need on campus for student leaders to take the lead in prevention of sexual violence. According to them, taking the lead will not only make others aware, but will be a way to help prevent sexual violence from happening in residences and around campus:

“*After I attended the workshops*, *I realized how much we needed to start … like discussion groups or like a sexual violence forum on campus where we as young men can talk about how we can strategize on how to prevent sexual violence from happening*”

**[Sitole, second-year Law student]**

*“The way I see it is that sexual violence can be prevented if we all play our part*, *men and women*, *but I think men play an important role in this and I also think as student leaders we can set the example for others*. *I also believe what we need here on campus is like an interest group for sexual violence … I mean like a space where we can discuss issues around sexual violence and how these issues can be addressed*”.

**[Posiso, third-year Medical student]**

These participants showed that they have been thinking about ways of preventing sexual violence after they attended the workshops. The positive influence of the workshops was reflected, and participants felt they needed to extend their experience and knowledge to others.

“Men with a conscience care about sexual violence prevention” emerged as a dominant theme from participants’ reflections following the fifth and sixth workshops. Participants reflected on their own realization that others’ views and opinions can influence one’s own values system, not necessarily in a negative way, but in a positive way too and that there was a definite need for men to engage. They related how men could engage with one another in dialogue around sexual violence prevention, where men’s attitudes and behaviour could be influenced positively.

Evidence of change was reflected six months after delivery of the intervention with five (5) participants, e.g.

*“I was thinking about this whole issue of sexual violence and i was chatting to a few guys in res after the workshops*, *even my brothers at home*, *that when we hear about how sexual violence is a reality in our society*, *that maybe we don’t want to face up to it coz it’s so scary … coz it can happen to anyone*. *I think if we as leaders can share our ideas and thoughts with others*, *like we did in the workshops … I think that will help make sexual violence a less scary issue to engage with”*.

*[Sitole*, *second-year Law student]*

Participants acknowledged that engaging in dialogues on sexual violence in general would take time, but that these dialogues were important in prevention of sexual violence. They acknowledged the fact that “we don’t want to face up to it coz it’s so scary”, but that through sharing ideas and thoughts, it “will help make sexual violence a less scary issue to engage with”.

Evidence of impact at cognitive level was reflected when one participant articulated how prevention of sexual violence was about “doing the right thing” and “respect”. He showed how he has engaged in thought:

“*In a way … uhm … the way I see it*, *is that this whole sexual violence thing and consent to sex has become an issue of what is right and what is wrong*. *I almost want to say that there is no in between or no grey area as we like to say*. *For me it has become what is the right thing to do and it’s about respect*”

**[Morgan, third year Commerce student]**

It is clear that the intervention assisted in participants’ learning about sexual violence, which resulted in a change in perceptions and confidence in talking about sexual violence.

## Discussion

The findings demonstrate how male student leaders in one university in South Africa engaged positively in a process of change over six workshops. Prevention of sexual violence was never considered part of their portfolio as student leaders, but the qualitative data from the intervention sessions showed their increasing accountability to prevent sexual violence.

Data from the interviews conducted six months post intervention delivery with five (5) participants, reflected the impact of the intervention and how participants have changed in their thinking about sexual violence. There were six important areas of influence and change were identified as follows: 1) *Change at a cognitive level 2) Change in relation to family and friends 3) the need to change their role in intimate relationships 4) Taking on the role as bystander 5) Sharing sexual violence prevention on social media 6) The need to engage in prevention intervention*.

A change in attitude was demonstrated when participants used the construct of consciousness in the naming of the adapted intervention. This makes the design of the Men with Conscience model unique, because it enables a community of university student leaders to start from nothing or limited knowledge to owning the problem [32]. For example, participants learned to make connections from what seemed trivial and unimportant, such as ***“cat whistling”*** and the notion of ***“gentle persuasion”*** to engage in sexual acts [e.g. 33], to the realization of how rape was a reality in the university environment and student leaders had a role to play in prevention of sexual violence in university residences.

This study showed the potential of tailored interventions that focused on gender identity in relation to the young men’s social norms and values, which were key components. The young age of perpetration underscores the need to intervene early in the life-course since it has the potential to have an impact on other drivers of violence against women i.e. alcohol misuse [34] In this study we adapted and intervention that target community settings with men of all ages [16], but have shown an adapted version can be developed for young men. Although the adolescent period is seen as the period for exponential risk taking associated with peer pressure and for habituation, it is also seen as a period for developing value systems. We saw in this study how peer norms and pressure remain key stressors for the young men and their questioning of cultural and gender value systems. There is therefore no doubt that working with young men could have long term gains for prevention. This supports findings from studies mainly from developed settings that showed primary prevention strategies with males can be effective in reducing rape myths and changing attitudes around consensual sex [35–40]. Studies in South Africa with high school learners show similar impact among adolescent boys with a decrease in perpetration reported [41, 42].

A very clear shift in perceptions and understanding was observed during the third workshop when participants unpacked rape and consensual sex. The recognition that rape is part of their own interactions with intimate partners and the role of social norms was revealing and led to a shift for participants during the intervention, which demonstrate a critical stage of self-reflection. The turning point reflected change, mainly at the individual and interpersonal levels of risk in the socio-ecological framework, but which could have subsequent impact on the community and social levels of risk [43].

The adaptation process to the Men with Conscience intervention demonstrated how the targeted six workshops assists males to reflect on their own values and belief systems, community norms and cultural practices that reinforce gender inequality and male dominance over women. Promoting healthy relationships and teaching men about healthy models of masculinity, have been shown to be critical and should remain the foundations of the Men with Conscience intervention. This also responds to the call for an increase in research on theory-based gender transformative interventions [44–46] through public action and advocacy for gender justice. The latter is in line with the SDG’s to address gender equality and is in the interest of the global sexual violence prevention agenda [47].

The Men with Conscience intervention thus, presents important mechanisms (pathways) of change. By gaining knowledge on sexual violence, through **dialogue** and discussion, men are challenged to start thinking more critically about sexual violence, for example, men’s personal values and belief systems and how this is influenced by societal pressures for men’s behaviour. **Reflexivity** enabled men to engage in their own construction of manhood, intimate relations and sexual violence. **Conscientization, an important pathway to change,** lead to change in men’s behaviour in their personal lives and social roles. There was also evidence of young men **taking ownership** of their own behaviour towards women and wanting to change.

Working with student leaders in higher education settings have limitations. For example, this sample of male students were all attending university which implies they had a reasonable high level of education and education has been shown to be protective against perpetration of violence against women [48, 49]. In addition, they were student leaders who may have volunteered for these positions and which implies leadership value systems. They may therefore, be different to the average university student. However, having participants with leadership qualities was also useful for the adaptation process. As leaders, they had insight into the students and their behaviour and they were able to engage in dialogue on sexual violence more than average male students. Their understanding of the University social systems was also proved to be helpful in developing interventions in the last workshop. Another study limitation was the focus on sexual violence. It is well known that in intimate partner violence, sexual violence seldom happens in isolation [50]. However, it is possible that the Men with Conscience intervention can bring about change in all forms of violence, but this would need to be tested.

This study was conducted in one university on student leaders, in university residence, a group who volunteered to participate in the study, which are limitations. Another limitation is that only five of the 15 participants were interviewed six months post intervention delivery. Although qualitative studies are by nature very subjective, adapting the One Man Can intervention to university settings was an important alternative, which this study has potential to show. The current evidence on interventions with young men is not conclusive and more work is needed. Interventions with women are needed, and recent studies have shown that interventions must be multifaceted with both men and women [51]. However, intervention development and delivery are not only costly, but also time consuming [52].

## Conclusion

This paper reflected on the adaptation of the OMCI, used in community settings, to the MWC intervention adapted for use in university settings. The process of adapting the One Man Can into the Men with Conscience Intervention showed the huge need to address sexual violence within the university, with young men eager to learn and skill themselves to fill the huge gap and become active in the prevention of sexual violence. What resonated in the adaptation was young men’s need for dialogue and their belief in the role they had to play in preventing sexual violence in the university setting.

Men with Conscience is a theory-based intervention. This model was designed for implementation specifically in university settings, and thus presents a critical response to the problem of sexual violence faced in universities in South Africa and the rest of the African continent. This places the Men with Conscience intervention ready for more rigorous evaluation as the next step to understanding its effectiveness with students to prevent sexual violence in South African universities.

## Supporting information

S1 Appendix Men with conscience manual. (DOCX)

S2 Appendix Post-intervention focus group guide. (DOCX)
