# Supplementary material for: “As young men we have a role to play in preventing sexual violence”: Development and relevance of the men with conscience intervention to prevent sexual violence
Source: PLoS One. 2021 Jan 7;16(1):e0244550. doi: 10.1371/journal.pone.0244550 (PMC7790258; doi:10.1371/journal.pone.0244550)
Supplement: S1 Appendix — (DOCX) [file pone.0244550.s001.docx]

**S1 Appendix:** **Men with Conscience Manual**

Workshop 1: Locate sexual violence in a university context

| Locating context | Content |
| --- | --- |
| Objective:  To assess the nature and social context of sexual violence in student residences  Materials:  - A space to conduct a group  - Chairs in a circle  Time:  45 minutes | Steps:   1. Arrange chairs in a circle for participants to engage in discussion, facing each other and the facilitator. 2. Allow participants to introduce each other, introduce the facilitators and the objective of the study. 3. Explain to participants the objective of the focus group today and the flow of events. 4. The session will start with a question, followed by more questions that will guide the discussion. 5. Guiding questions for discussion: (also see Appendix H).  - Have you ever participated in any intervention on sexual violence? If so, which one? - When thinking about your university environment, what factors do you think are contributing to or causing sexual violence? - What factors do you think protect against sexual violence? - What do you think a plan for preventing sexual violence in your university should include? - Who else do you think we should talk to or interview?   Facilitator’s notes:  The currents statistics on sexual violence in South Africa are rising, with no evidence of any decline. Sadly, the university environment as part of the wider society is also affected by this. This pre-focus group discussion is a good way to assess where the young male student leaders are at and how they view and experience sexual violence within the student residence system. |

**Workshop 2: Personal values and belief systems**

| MWC | Content |
| --- | --- |
| Theme/Title:  Personal values and belief systems  Objective:  To explore values and attitudes around sexual violence and men  Materials:  1. Four signs  “Strongly Agree”  “Strongly Disagree”  “Agree”  “Disagree”  2.Flip chart paper (to jot comments)  Time:  45 minutes | Steps:   1. Before the activity begins, put up the four signs around the room, leaving enough space between them to allow a group of participants to stand near each on. Review the statements provided in the facilitator’s notes section and choose five or six that you think will lead to the most discussion. 2. Explain to the participants that this activity is designed to give them a general understanding of their own and each other’s values and attitudes about gender. Remind the participants that everyone has a right to his or her own opinion, and no response is right or wrong. 3. Read aloud the first statement you have chosen: “It is easier to be a man than it is to be a woman”. Ask participants to stand near the sign that says what they think about the statement. After the participants have moved to their sign, ask for one or two participants beside each sign to explain why they are standing there and why they feel this way about the statement. 4. After a few participants have talked about their attitudes towards the statement, ask if anyone wants to change their mind and move to another sign. Then bring everyone back together and read the next statement: “women who wear short skirts invite rape”. Repeat steps 3 and 4. Continue for each of the statements that you chose. Other statements are:  - Sex is more important to men than to women” - If a man is sexually aroused it is very difficult for him not to have sex”  1. After discussing all of the statements, lead a discussion about values and attitudes about gender by asking these questions:  - Which questions or statements were difficult to give an opinion on, if any? - Why was it difficult to give an opinion on certain statements? - When a man is sexually aroused, do you think it plays a role in sexual harassment or rape? |

**Workshop 3: Societal pressures for men’s behaviour**

| MWC | Content |
| --- | --- |
| Theme/Title:  Societal pressures for men’s behaviour  Objective:  Materials:  1) Flip chart paper  2) Markers  3) Koki pens  Time:  60 minutes | Steps:   1. Divide participants into two groups of at least 6 participants per group. 2. Give each group a sheet of flip chart paper and koki’s for writing. 3. The one group has to discuss the following question: What does it mean to be a man? 4. The second group has to discuss the following question: What expectations do society place on us for being men? 5. Give participants 20 minutes to discuss these questions in their respective groups then allow each group to share their thoughts and views to the larger group. 6. Based on the issues raised, lead a discussion around the important issues. 7. Issues raised may include the following:  - society prescribes how men should behave - women are not respected - the influence of social values and norms  1. Conclude the discussion by summarizing important points raised. |

**Workshop 4: Defining Rape**

| MWC | Content |
| --- | --- |
| Title:  Defining Rape  Objective:  To inform participants on what it means to have consensual sex  Materials:  Time:  60 minutes | Steps:   1. Divide the larger group into smaller groups of 3 each. 2. Give each participant a copy of the legislation on Rape. 3. Give each group a case scenario based on real life events – (see cases for discussion below) 4. Allow each group 10 minutes to read the scenario and discuss the questions at the end of the scenario, with reference to the legislation on rape. 5. After participants in each group read the scenarios and discussed their responses, ask each group to share their case scenario and their responses to each of the questions raised in the scenario. 6. Continue this exercise until all groups participated and shared their views with the larger group.   Cases for discussion:   1. Two gay men drinking at a bar and they discuss having sex. 2. A man and a women engaging in sex and the women decides midway she doesn’t want to continue. 3. A women drinks with a guy at a bar and wakes up in his bed the next day. 4. In closing, summarise each group’s responses in light of what they think rape is in relation to the legislation. |

**Workshop 5: Courage to act**

| MWC | Content |
| --- | --- |
| Theme/Title:  Courage to act  Objective:  To explore men’s perceptions of their degree of courage in bystander intervention  Materials:  Time:  60 minutes | Steps:   1. Participants seated with chairs in a circle facing each other and the facilitator. 2. Inform participants that the session will be looking at their level of courage to intervene in a situation they feel is abusive. 3. Give each participant a scenario which they need to read and decide which level of courage it would take to intervene, e.g. least courage, some courage or most courage. 4. Put these headings, Least courage, Some courage or Most courage on a board. 5. Ask participants the following question: “Based on your knowledge you’ve gained the last few weeks, decide how you will intervene if you will intervene, e.g. least courage, some courage or most courage to intervene. 6. Ask each participant to put his scenario under the level of courage he felt it would take to intervene. Scenarios were:  - If there is a conflict in a marriage - Men joking about women’s clothing, what would you do? - If your friend is abusing his girlfriend   Facilitator’s notes:  This activity looks at bystander intervention and helps participants to consider their role in bystander intervention. It looks at the level of courage participants think it would take to intervene in certain scenarios related to sexual violence perpetration. |

**Workshop 6: Maintaining your own identity in intimate relationships**

| MWC | Content |
| --- | --- |
| Theme/Title:  Maintaining your own identity in intimate relationships  Objective:  To be able to identify healthy vs and unhealthy relationship  Materials:  A space for discussion  Time:  60 minutes | Steps:  1. Put up 3 signs up with the following headings: Healthy, Unhealthy & Depends  2. Put chairs in a circle and seat participants in a circle.  3. Explain to participants that today’s session will look at relationships.  4. Question for discussion:   - If you think of relationships, what do you think are some of the qualities of a healthy relationship?   Some qualities:  Communication  Trust  Openness  5. Give each participant a statement. Statements were:  - One person hits the other in order to have this person obey him or her  - Sex is not talked about  - You are in control and you are able to do what you want to so  - You argue & fight often  - You stay in the relationship because it is better than being alone  - You will do anything for your partner  - One person usually makes every decision for the couple  - Your partner is still close to his or her ex-boyfriend or ex-girlfriend    6. Let each participant put a statement under a heading he thinks fits the statement.  7. Tell participants to scan through these & if you feel any of these statements need to move then move and let’s discuss.  8. Let all participants have a look at what each other put where and lead the discussion in cases where different choices were made.  9. Repeat 6 – 8 for each question.  Facilitator’s notes:  This activity looks at how men perceive healthy versus unhealthy relationships and how they think they will respond in each case. What their reactions will be in case of sexual abuse, either personally or as bystanders/observers of abuse. |
