# Supplementary material for: “As young men we have a role to play in preventing sexual violence”: Development and relevance of the men with conscience intervention to prevent sexual violence
Source: PLoS One. 2021 Jan 7;16(1):e0244550. doi: 10.1371/journal.pone.0244550 (PMC7790258; doi:10.1371/journal.pone.0244550)
Supplement: S2 Appendix — (DOCX) [file pone.0244550.s002.docx]

**S2 Appendix:** **Post-intervention focus group guide**

You have come to the last phase of this study and thank you for your commitment and valuable contributions you have made towards this study and the development of the intervention to prevent sexual violence in university residences.

We would like to find out how you experienced this journey.

1. How would you describe your experience after attending all these sessions?
2. What was the “take home” message for you after attending the sessions every week?
3. If there was anything that you could change, what would that be?
4. Are there any other aspects that you feel need to be addressed? If so, with whom?
